# Confirmation of Paternity despite Three Genetic Incompatibilities at Chromosome 2

**DOI:** 10.3390/genes12010062

**Published:** 2021-01-04

**Authors:** Andrzej Doniec, Wojciech Łuczak, Maria Wróbel, Miłosz Januła, Andrzej Ossowski, Paweł Grzmil, Tomasz Kupiec

**Affiliations:** 1 Department of Genetics and Evolutionism, Institute of Zoology and Biomedical Research, Jagiellonian University, Gronostajowa 9, 30-387 Kraków, Poland; andrzej.doniec@uj.edu.pl; 2 Forensic Genetics Section, Institute of Forensic Research, Westerplatte 9, 31-033 Kraków, Poland; mwrobel@ies.krakow.pl (M.W.); mjanula@ies.krakow.pl (M.J.); 3 Laboratory of Molecular Diagnostics Genmed, ul. Św. Marcin 49, 61-806 Poznań, Poland; wluczak@gmail.com; 4 Molecular Biology Techniques Laboratory, Faculty of Biology, Adam Mickiewicz University Poznań, Umultowska 89, 61-614 Poznan, Poland; 5 Department of Forensic Medicine, Pomeranian Medical University of Szczecin, Powstańców Wielkopolskich 72, 70-111 Szczecin, Poland; forensicmedicine1978@gmail.com

**Keywords:** uniparental disomy, paternity testing, trisomy rescue, short tandem repeat profiling

## Abstract

DNA testing in cases of disputed paternity is a routine analysis carried out in genetic laboratories. The purpose of the test is to demonstrate similarities and differences in analyzed genetic markers between the alleged father, mother, and a child. The existence of differences in the examined loci between the child and the presumed father may indicate the exclusion of biological parenthood. However, another reason for such differences is genetic mutations, including chromosome aberrations and genome mutations. The presented results relate to genetic analyses carried out on three persons for the purposes of disputed paternity testing. A deviation from inheritance based on Mendel’s Law was found in 7 out of 53 STR-type loci examined. All polymorphic loci that ruled out the paternity of the alleged father were located on chromosome 2. Additional analysis of 32 insertion–deletion markers (DIPplex, Qiagen) and sequencing of 94 polymorphic positions of the single nucleotide polymorphism (SNP) type (Illumina, ForenSeq) did not exclude the defendant’s biological paternity. A sequence analysis of STR alleles and their flanking regions confirmed the hypothesis that the alleles on chromosome 2 of the child may originate only from the mother. The results of the tests did not allow exclusion of the paternity of the alleged father, but are an example of uniparental maternal disomy, which is briefly described in the literature.

## 1. Introduction

DNA testing in disputed paternity cases is a routine analysis carried out in forensic laboratories. The standard procedure consists of comparison of STR marker variants in the child, the mother, and the alleged father. Development of the massively parallel sequencing technique shows the high usefulness of SNP (single nucleotide polymorphism) analysis for interpretation of the degree of kinship, especially when any aberration event (such as mutation) is involved [1,2,3]. A basic assumption of genetic analysis in disputed paternity cases is that the child inherits half of its DNA from each of its biological parents. Lack of common alleles in examined loci between the child and the alleged father may indicate paternity exclusion of the examined man. However, genetic mutations—particularly chromosome aberrations and genomic mutations—may also result in a lack of compatibility [4,5,6].

The phenomenon of uniparental disomy (UPD) is a rarely occurring genomic mutation (with a different frequency of occurrence for each chromosome) [7], which is at the same time an example of non-Mendelian inheritance. In UPD cases, the child receives two copies of a given chromosome or fragments of a chromosome from only one of the parents without a copy originating from the second. UPD can take the form of heterodisomy, where a pair of non-identical chromosomes is conveyed by one of the parents (error during meiosis I) or isodisomy, where an individual chromosome from one of the parents is duplicated (error during meiosis II). The most common mechanism causing UPD is loss of one of the chromosomes from an initially trisomic zygote that arises as a result of a meiotic nondisjunction event. Uniparental disomy may manifest itself phenotypically in physiological disorders as a result of disturbance of parental imprinting or by the occurrence of monogenic diseases because of heterozygosity loss or chromosomal mosaicism. The occurrence of maternal UPD has been linked to genetic diseases, including Prader–Willi syndrome and Angelman syndrome [8,9,10].

This article describes an example of a paternity case where analysis of a set of standard STR markers showed a lack of inheritance of common alleles between the alleged father and the child in markers located exclusively on chromosome 2. The location of genetic markers that excluded the paternity of the alleged father on one chromosome strongly indicated a hypothesis of maternal uniparental disomy of chromosome 2. Standard STR analysis was extended by a panel of SNP markers and insertion–deletion (in-del) markers. Additionally, sequencing analyses of nuclear DNA STR markers using next-generation sequencing were performed. In order to determine the basic type of disomy, we analyzed an additional six STR loci on chromosome 2, which were selected on the basis of data in the 1000 Genomes Project database.

## 2. Materials and Methods

At the time of buccal swab collection, no phenotype disturbances were found that could be attributed to UPD or could be partially the result of UPD of chromosome 2.

### 2.1. DNA Samples

Samples for paternity tests were collected from swabs of the inner cheek epithelium from 3 examined subjects: the alleged father, the daughter, and the mother. DNA extraction was performed with the Swab kit (A&A Biotechnology, Gdynia, Poland), according to the manufacturer’s protocol.

### 2.2. Selection and Analysis of New STR Markers on Chromosome 2

On the basis of the 1000 Genomes database (https://www.internationalgenome.org/) and the STRcat web application (http://strcat.teamerlich.org), we selected 6 sites with STR characteristics, located on both arms of chromosome 2. One of the regions (D2N43) has been previously reported in the literature [11]. For the remaining selected regions, pairs of primers and analysis conditions were designed. Pre-selected loci were tested for the degree of heterozygosity in the Polish population (Table 1).

List of primers used in this study:

1. D2N43—FP: 6FAM-5′TTAA TAAA TGCA CTCA CACT CTAG ATAG, RP: 5′GTTC CAGG AGCA TCTC CATC C3′;
2. D2L221—FP: 6FAM-5′CATC CAGC AGGA TTTC TTTC T, RP: TGTG AGCT ATGA TTAT GCCA TTG3′;
3. D2L142—FP: 6FAM-5′CATT GAAA TAAT TAAC CTCA CATT TTC 3′, RP: 5′GAAA CTAA ATGT CAGT TGTT TTC 3′;
4. D2L174—FP: TAMRA-5′CGGA TACA CACC ACTG TTGG AC3′, RP: 5′AGCC AGAA AAGC ATAC CCGT3′;
5. D2L114—FP: HEX-5′GATA AATT CCAC TCTT GGTC ATAT AC3′, RP: 5′GTCA GTGC TAGA GGCA TATT TACA3′;
6. D2L234—FP: ROX-5′AAAG TGCA AGGT TTGA AGCC3′, RP: 5′AGCT GTGG TTGG CGAT CATT3′;
7. D2L231—FP: 6-FAM-5′AGGC TGAT CATT TGAC TTTC TTTG T3′, RP; 5′CACA TGCT CTCA CTCA TAAG TGGA3′.

#### 2.2.1. PCR Reaction

PCR reactions were performed with the Type-it Multiplex PCR Master Mix kit (Qiagen, Hilden, Germany), with 0.2 µM of each primer and 1 ng of template DNA to produce a final volume of 10 µL. The following thermal profile was used for all designed primers: 95 °C for 5 min, followed by 30 cycles of 95 °C for 30 s, 60 °C for 90 s, 72 °C for 30 s; final extension at 65 °C for 30 min.

#### 2.2.2. Electrophoresis

PCR products were prepared for electrophoretic separation by adding 1 µL of product to 9 µL of GeneScan 600 LIZ dye Size Standard v2.0 DNA (Applied Biosystems, Foster City, CA, USA) with formamide. Electrophoresis was performed with an ABI PRISM 3500xL Genetic Analyzer (Life Technologies, Carlsbad, CA, USA), using a POP-4 polymer. Samples with designed primers were run on an ABI 3130xL (Life Technologies, USA) with a POP7 polymer. Data analysis was performed with GeneMapper ID-X ver. 1.4 (Life Technologies, USA).

### 2.3. Quantification and PCR-CE Analysis with Commercially Available STR and In-Del Kits

DNA was quantified with the Quantifiler Trio DNA Quantification Kit (Life Technologies, USA) with a 7500 PCR Real Time System (Applied Biosystems, USA). Samples were amplified (with 1 ng targeted DNA input) using an NGM kit (Life Technologies, USA), GlobalFiler kit (Life Technologies, USA), Investigator DIPplex kit (Qiagen, Hilden, Germany), and Investigaror HDplex kit (Qiagen, Hilden, Germany), and run on a 3500xL Genetic Analyzer for Human Identification (Life Technologies, USA). Electrophoresis results were analyzed using GeneMapper IDx ver. 1.5 software (Life Technologies, USA). Blanks and control 007 DNA (0.1 ng/μL) (Life Technologies) were used as a quality control.

### 2.4. Next Generation Sequencing

Sequencing was performed using the Illumina MiSeq FGx Forensic Genomics System (Illumina, San Diego, CA, USA) and ForenSeq kit (Illumina, USA), according to the manufacturer’s protocol. Data analysis was performed using UAS (Universal Analysis Software, Illumina).

### 2.5. Statistical Analysis

Statistical analysis of the commercially available STR marker set was performed using GenoProof 3 (Qualitype, Dresden, Germany).

## 3. Results

**Figure 1:**
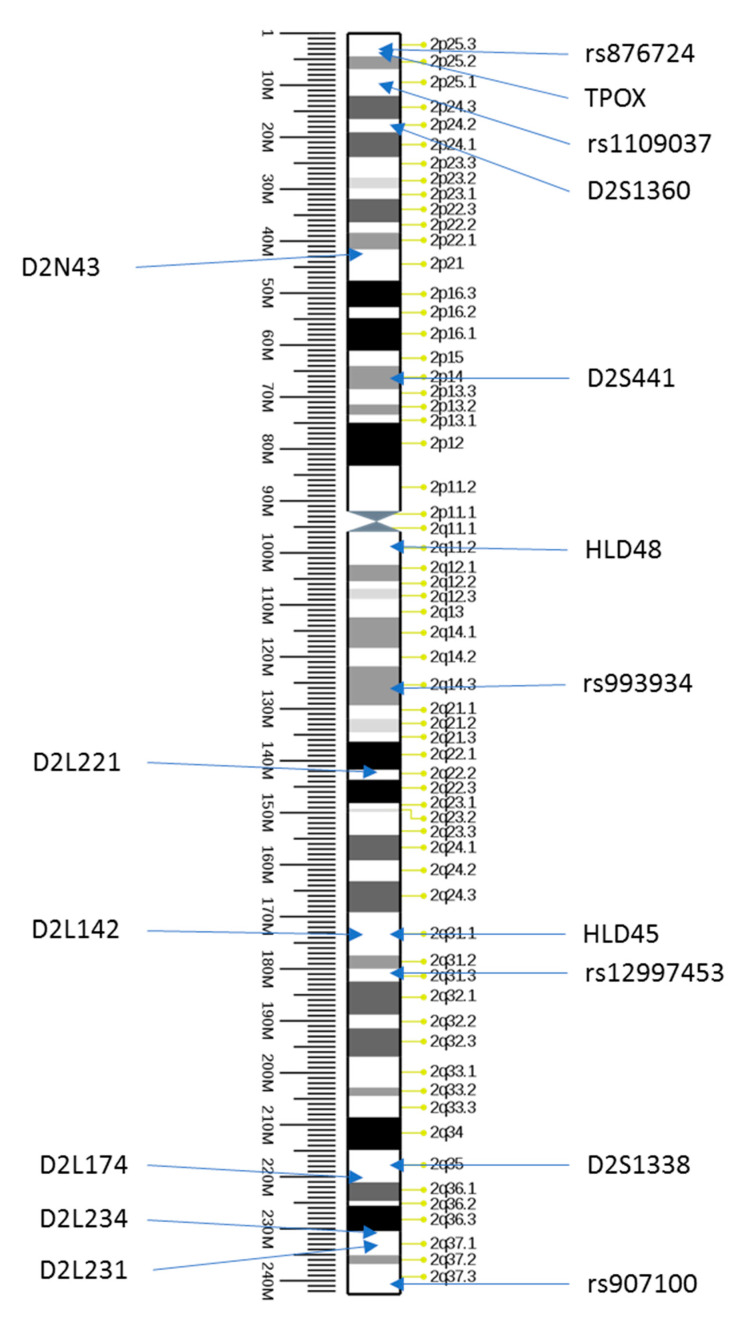
Structure of human chromosome 2 with indicated loci of tested markers at chromosome (source: Ensemble, modified).

The above-described paternity test, carried out using 47 standard STR markers of a commercially available STR kit (Appendix A), followed by PCR-CE analysis, showed that, except for TPOX, D2S441, and D2S1338 markers located on chromosome 2 (Figure 1, Table 2), the results of all other markers confirmed the paternity of the alleged father (Appendix A). A statistical approach requires use of STR databases that are well described in the literature, characterizing the local population [12,13]. For this reason, statistical analysis of the paternity index (PI) was carried out on 24 markers included in the Globalfiler kit, and allele frequencies for the Polish population with a mutation rate of 0.001 (mutation model: equal mutation probabilities with increasing range) gave a result of Combined Paternity Index (CPI)= 0.0866, which excludes the hypothesis of the defendant’s biological paternity. Analysis of the incompatible loci—TPOX, D2S441, and D2S1338 in samples from the child, alleged father, and mother—revealed the compatibility of the alleles of the mentioned markers between the mother and the child. Sequencing of these three markers together with flanking regions located at chromosome 2, using Massively Parallel Sequencing (MPS) technology, confirmed the results obtained by PCR-CE analysis (Table 2). Moreover, sequencing of the D2S441 locus of the homozygous child with the 11.3 variant revealed a single nucleotide deletion of thymidine (T) in the fifth repetition of the (TCTA) motif. An identical deletion was present in the mother’s sequence, who was a heterozygote 11.3/14 at this locus, while the alleged father had alleles 11 and 15 of the D2S441 locus without a characteristic deletion in the repetitive motif (Table 3). This result suggested a hypothesis of the maternal origin of both chromosomes 2 of the child, and thus occurrence of maternal uniparental disomy (UPD2).

The analysis of additional markers including 170 SNP and 7 X-chromosome STR markers with the use of MPS technologies and additionally 30 in-del polymorphisms by use of standard PCR-CE methods was performed to evaluate this hypothesis. The results supported the existence of UPD for chromosome 2 and explained the incompatibility of markers between the child and the alleged father and thus did not rule out the biological paternity of the tested man (Appendix A). Calculations of PI (Globalfiler kit) performed with the exclusion of the loci on chromosome 2 showed extremely strong support for the hypothesis of biological paternity, giving CPI = 6.35 × 10^7^ [14].

Furthermore, the results of analysis of all markers located on chromosome 2 revealed that the uniparental disomy of this chromosome could be accompanied by two crossing over events. The first recombined region spanned the sequence between TPOX and D2L142 loci and the second region was located distally from the D2L234 locus. This finding explains the homozygosity of the child in the rs1109037, D2S441, rs993934, D2L221, and D2L231 loci (the mother was heterozygous in all these markers).

Table A1 in Appendix A summarizes the STR results obtained using different kits (47 markers). Excluding markers located at chromosome 2, no evidence was found to suggest that the alleged father can be excluded. Subsequent NGS, SNP (Table A2), and in-del (Table A3) analysis under the same conditions strongly supported the biological paternity hypothesis.

## 4. Discussion and Conclusions

This article describes an example of a paternity case where standard STR markers analysis showed a lack of inheritance of common alleles between the alleged father and the child in loci located exclusively on chromosome 2. A hypothesis was put forward not to exclude the man as the father but to explain the location of the genetic markers, which excluded the paternity of the alleged man on one chromosome by maternal uniparental disomy of chromosome 2. Standard STR analysis was extended by a panel of SNP markers and insertion–deletion (in-del) markers. Additionally, sequencing analysis of nuclear DNA STR markers using massive parallel sequencing was performed. In order to determine the basic type of disomy, we analyzed an additional six STR loci selected from the 1000 Genome Project database on chromosome 2.

This is an interesting paternity case where standard STR marker analysis indicated exclusion of paternity of the alleged father, while more in-depth analysis of chromosome 2 located markers explained the inconsistency of the obtained standard results by maternal uniparental disomy (mUPD) of chromosome 2. The analysis of additional 17 STR, in-del, and SNP markers located on both arms of chromosome 2 allowed exclusion of segmental mUDP and confirmed complete mUDP. In addition, the region of two probable crossing over events was narrowed down.

While UPD is a rare event [15,16], in particular paternity cases, it might complicate the interpretation of paternity index (PI) statistics. This phenomenon might affect any chromosome [17,18,19], and in the worst-case scenario may lead to false exclusion of kinship. Thus, in paternity cases, more attention should be paid when loci that do not conform to Mendelian inheritance rules are restricted to a single region or chromosome. The combination of several molecular techniques, i.e., STR analysis, SNP array, and massively parallel sequencing, was demonstrated to be a very effective strategy in identification of UPD [20]. Although UPDs in paternity testing have been described for small chromosomes [21,22], few cases of maternal uniparental isodisomy of chromosome 2 have previously been reported [19,23]. UPD can hinder paternity or maternity tests but might also influence database queries in identification of missing persons. The UPD incidence was estimated to be 1 in 3500 live born babies [15], but this frequency might be underestimated as most reports present cases with a clinical phenotype [7]. Our result gives an additional example of UPD, and together with previous reports indicates the strong need to extend the panel of molecular markers analyzed to avoid false exclusions of relationships in forensic genetics [20,21,22,23].

The child from our report demonstrated homozygosity in five loci (rs1109037, D2S441, rs993934, D2L221, and D2L231) on chromosome 2, but the mother was heterozygous in all these five loci. On the other hand, in four other loci (TPOX, D2L142, rs12997453, and D2S1338) on chromosome 2, the child was heterozygous like the mother. TPOX is located distally to rs1109037, and D2L142 and D2S1338 are located between D2L221 and D2L231. The most probable explanation is that two crossing-over events occurred between maternal chromosomes. Although UPD was described in the 1980s [8,9], the molecular mechanism underlying this phenomenon is still not very well understood. UPD might appear due to gamete complementation, monosomic or trisomic rescue, or mitotic or meiotic errors [7,23]. In the child analyzed in this work, we suggest that the error occurred during the first meiotic division as the occurrence of two crossing-over events is the most probable explanation of the observed alleles at chromosomes 2 in the mother and child. Since UPDs might result due to different mechanisms, an individual analysis of the genetic background of each UPD case is very important not only from the medical point of view, but also in forensic genetics.

In conclusion, UPD might be challenging for routine analyses in forensic laboratories and should be taken into consideration to avoid a false exclusion of a relationship and to obtain a confident kinship conclusion.

## Figures and Tables

**Table 1:** Characteristics of selected STR type markers located on chromosome 2 together with the results of their examination for the alleged father, child, and mother. The chromosome location, repeat motif, heterozygosity (based on strcat.teamerlich.org), and arbitrary size in bp are indicated. Underlined genotypes show a lack of inheritance of common alleles between the alleged father (AF) and child.

Name	Repeated Motif	Heterozygosity	Location	AF	Child	Mother
D2N43 *	(AGAT)	n/a	42,072,419—42,072,494	458; 470	498	498
D2L221	(AAAG)	0.864	142,567,661—142,567,756	259; 279	259	239; 259
D2L142	(AAAG)	0.887	174,327,360—174,327,425	410; 430	424; 438	424; 438
D2L174	(AAAG)	0.911	221,218,006—221,218,073	175; 189	193	193
D2L234	(AAAG)	0.878	231,153,171—231,153,239	244; 253	244; 248	244; 248
D2L231	(AAAG)	0.883	234,570,270—234,570,328	141; 162	159	159; 154

* marker described previously by Novroski et al. (2018) [11].

**Table 2:** Genotypes of the alleged father, mother, and child at different markers located on chromosome 2 and analyzed by commercially available kits. Underlined genotypes show a lack of inheritance of common alleles between the alleged father (AF) and child.

Kit	Maker Type	Name	Location	AF	Child	Mother
ForenSeq	SNP	rs876724	2p25.3	C,C	C,C	C,C
ForenSeq	SNP	rs1109037	2p25.1	A,A	A,A	G,A
ForenSeq	SNP	rs993934	2q14.3	T,T	T,T	T,C
ForenSeq	SNP	rs12997453	2q31.3	G,G	A,G	A,G
ForenSeq	SNP	rs907100	2q37.3	G,C	C,C	C,C
DIPplex	indel	HLD45	2q31.1	−,+	+,+	+,+
DIPplex	indel	HLD48	2q11.2	−,−	−,−	−,−
GlobalFiler	STR	TPOX	2p25.3	8,8	9,11	9,11
HDplex	STR	D2S1360	2p24.2	22,22	22,22	22,22
GlobalFiler	STR	D2S441	2p14	11,15	11.3,11.3	11.3,14
GlobalFiler	STR	D2S1338	2q35	18,18	21,23	21,23

**Table 3:** Allele nucleotide sequences of marker D2S441 with highlighted single nucleotide deletion.

POI	Allele	Nucleotide Sequence of Allele
Daughter	11.3	(TCTA)_4_ TC 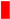 A (TCTA)_7_
	11.3	(TCTA)_4_ TC 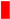 A (TCTA)_7_
AF	11	(TCTA)_11_
	15	(TCTA)_15_
Mother	11.3	(TCTA)_4_ TC 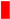 A (TCTA)_7_
	14	(TCTA)_14_

## Data Availability

The data presented in this study are contained within the article.

## Appendix A

**Table A1:** Summary of obtained STR genotypes using NGM, GlobalFiler, HDplex, and NGS (AF—alleged father). The four bolded loci are located at chromosome 2 and indicate abnormalities in Mendelian inheritance.

Locus	PF	Daughter	Mother
Amelogenin	X,Y	X,X	X,X
D1S1656	16, 19.3	16, 17.3	16, 17.3
**TPOX**	**8, 8**	**9, 11**	**9, 11**
**D2S441**	**11, 15**	**11.3, 11.3**	**11.3, 14**
**D2S1338**	**18, 18**	**21, 23**	**21, 23**
D3S1358	18, 18	17,18	17, 18
D4S2408	8, 9	8, 9	8, 12
FGA	20, 21	20, 21	21, 26
D5S818	12, 12	9, 12	9, 10
CSF1PO	10, 10	10, 10	10, 10
D6S1043	11, 19	11, 19	11, 11
D7S820	11, 12	9, 12	9, 10
D8S1179	10, 14	10, 14	8, 10
D9S1122	13, 13	12, 13	12, 12
D10S1248	13, 17	13, 13	13, 14
TH01	6, 7	6, 9.3	9, 9.3
vWA	16, 16	16, 18	17, 18
D12S391	18, 19	19, 22	21, 22
D13S317	8, 8	8, 8	8, 12
PentaE	11, 17	14, 17	12, 14
D16S539	12, 13	11, 12	11, 11
D17S1301	11, 13	12, 13	12, 13
D18S51	15, 18	17, 18	13, 17
D19S433	13, 14	13, 14	14, 15
D20S482	11, 14	11, 13	13, 15
D21S11	29, 31.2	28, 29	28, 32.2
PentaD	12, 13	12, 16	10, 16
D22S1045	11, 16	16, 16	15, 16
DYS391	10	-	-
SE33 (actB)	26.2, 27.2	18, 27.2	18, 27.2
DXS10135	26	26	22, 26
DXS8378	13	13	11, 13
DXS7132	15	12, 15	12, 14
DXS10074	19	8, 19	8, 17
DXS10103	19	19, 20	20, 21
HPRTB	12	12	12
DXS7423	15	15	14, 15
Penta E	11, 17	14, 17	12, 14
**D2S1360**	**22**	**22**	**22**
D3S1744	14, 18	18	16, 18
D4S2366	13, 14	9,14	9, 11
D5S2500	12	12, 16	16
D6S474	13	13, 14	14, 15
D7S1517	24, 25	24, 25	25
D8S1132	20, 21	20, 22	22, 23
D10S2325	7, 13	7, 13	8, 13
D21S2055	26, 29, 33	19.1, 29	19.1, 33

**Table A2:** Summary of single nucleotide polymorphism (SNP) genotypes obtained using Illumina MiSeq FGx Forensic Genomics System NGS (AF—alleged father). Five bolded loci are located at chromosome 2.

Locus	AF	Daughter	Mother
rs1490413	G,A	G,G	G,G
rs560681	A,G	A,A	A,A
rs1294331	G,A	A,A	G,A
rs10495407	G,A	A,A	G,A
rs891700	G,G	A,G	A,G
rs1413212	G,G	A,G	A,A
**rs876724**	**C,C**	**C,C**	**C,C**
**rs1109037**	**A,A**	**A,A**	**G,A**
**rs993934**	**T,T**	**T,T**	**T,C**
**rs12997453**	**G,G**	**A,G**	**A,G**
**rs907100**	**G,C**	**C,C**	**C,C**
rs1357617	A,A	T,A	T,T
rs4364205	G,G	T,G	T,G
rs2399332	A,A	A,C	C,C
rs1355366	A,G	A,G	A,G
rs6444724	T,C	C,C	T,C
rs2046361	A,T	A,T	T,T
rs279844	A,A	A,A	A,A
rs6811238	G,G	T,G	T,G
rs1979255	G,C	G,C	C,C
rs717302	G,A	A,A	A,A
rs159606	A,G	G,G	G,G
rs13182883	G,A	G,A	G,G
rs251934	T,C	C,C	T,C
rs338882	C,T	C,T	T,T
rs13218440	G,A	G,G	G,G
rs1336071	G,G	G,G	G,G
rs214955	G,A	A,A	A,A
rs727811	A,A	C,A	C,C
rs6955448	C,C	C,T	T,T
rs917118	C,T	C,C	C,C
rs321198	T,T	T,T	T,C
rs737681	C,C	C,C	C,C
rs763869	C,T	C,C	C,C
rs10092491	T,T	T,C	C,C
rs2056277	C,T	T,T	C,T
rs4606077	T,C	C,C	T,C
rs1015250	G,G	G,G	G,C
rs7041158	C,T	C,C	C,T
rs1463729	A,A	A,A	A,A
rs1360288	C,C	C,C	C,T
rs10776839	T,T	T,T	T,T
rs826472	C,C	T,C	T,T
rs735155	G,G	G,A	G,A
rs3780962	T,C	T,C	T,C
rs740598	A,A	A,A	A,A
rs964681	T,C	T,T	T,T
rs1498553	C,T	T,T	T,T
rs901398	T,T	T,T	C,T
rs10488710	C,C	C,C	G,C
rs2076848	T,T	T,A	T,A
rs2107612	A,A	A,A	A,A
rs2269355	C,G	C,G	G,G
rs2920816	C,C	T,C	T,C
rs2111980	G,G	G,G	G,G
rs10773760	A,A	A,A	A,G
rs1335873	T,T	A,T	A,T
rs1886510	C,T	C,T	T,T
rs1058083	A,G	G,G	G,G
rs354439	T,T	T,T	T,T
rs1454361	A,A	A,A	A,A
rs722290	C,C	C,G	C,G
rs873196	C,T	T,T	C,T
rs4530059	G,G	G,A	G,A
rs1821380	G,C	G,C	C,C
rs8037429	C,T	C,T	C,T
rs1528460	C,T	C,T	T,T
rs729172	C,C	C,C	C,C
rs2342747	G,G	A,G	A,G
rs430046	C,C	C,C	C,T
rs1382387	G,T	T,T	T,T
rs9905977	A,G	G,G	G,G
rs740910	A,A	A,A	A,A
rs938283	T,C	T,T	T,T
rs8078417	C,C	C,C	C,C
rs1493232	A,A	A,A	A,A
rs9951171	A,A	A,A	G,A
rs1736442	A,G	A,A	A,A
rs1024116	G,A	A,A	G,A
rs719366	T,T	C,T	C,C
rs576261	A,A	A,C	A,C
rs1031825	C,C	C,C	C,C
rs445251	C,G	G,G	C,G
rs1005533	G,A	G,A	G,A
rs1523537	T,C	T,T	T,C
rs722098	A,A	A,A	A,A
rs2830795	A,A	A,A	A,G
rs2831700	A,G	G,G	A,G
rs914165	A,A	G,A	G,A
rs221956	C,C	C,C	T,C
rs733164	G,G	G,G	G,G
rs987640	T,T	T,A	A,A
rs2040411	A,A	A,A	A,A
rs1028528	A,G	A,G	A,G

**Table A3:** Summary of obtained insertion–deletion (in-del) genotypes using DIPplex (AF—alleged father). The two bolded loci are located at chromosome 2.

Locus	AF	Daughter	Mother
Amel. X	X	X	X
Amel. Y	Y	−	−
HLD77	+	+	+
**HLD45**	**−/+**	**+**	**+**
HLD131	−/+	−/+	−
HLD70	+	+	+
HLD6	−/+	+	+
HLD111	−/+	−/+	−/+
HLD58	−/+	+	+
HLD56	−/+	−/+	+
HLD118	+	−/+	−/+
HLD92	+	−/+	−/+
HLD93	+	+	+
HLD99	+	−/+	−/+
HLD88	−	−	−
HLD101	−/+	−/+	−/+
HLD67	+	−/+	−
HLD83	+	−/+	−
HLD114	+	+	+
**HLD48**	**−**	**−**	**−**
HLD124	−/+	−/+	+
HLD122	−/+	−	−
HLD125	-	−/+	+
HLD64	-	−	−
HLD81	-	−/+	−/+
HLD136	+	+	−/+
HLD133	+	−/+	−/+
HLD97	−/+	+	+
HLD40	+	+	+
HLD128	−/+	−/+	−/+
HLD39	−	−	−
HLD84	+	−/+	−
